# Editorial – annus horribilis

**DOI:** 10.1017/ehs.2020.65

**Published:** 2021-01-12

**Authors:** Ruth Mace

**Affiliations:** London

## Abstract

**Figure:**
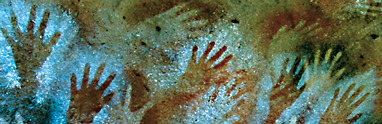

Because I talked about it in my last editorial, I will try not to talk too much about the microscopic elephant in the room. It floored me for a few weeks (darnn! I mentioned it already) but I have now recovered, mostly. I extend my sympathies to those whose life or loves have been damaged by this ruinous virus. And now I will try to talk about other things.

At the time of writing 1 January looms, which is a date of some consequence for the journal (as well as for us Brits, who are wondering if we are going to have to spend the next few years living almost exclusively on fish). This is the date that *EHS* will start charging authors for publication. This is how open access publishing works. The good news is that many of you may find that your institution is part of a Read and Publish agreement with Cambridge University Press – take a look: https://www.cambridge.org/core/services/open-access-policies/read-and-publish-agreements. If you are lucky enough to be at the University of Cambridge, University of California, any Max Planck Institute, most universities in Austria, The Netherlands, Switzerland and many more individual institutions, then you may find out that your institution has already paid for you to publish Gold Open Access in CUP journals, including *EHS*; and no doubt your universities are hoping you will make good use of their investment. CUP has a large number of such agreements under discussion and expect many more to be announced in 2021. If your institution is not on one of those lists (which mine is not yet), then do try to encourage your librarians to consider signing up to one of these transformative agreements. I am hopeful that this may be a route by which open access publishing can flourish. If you are from a very poor country your fee will be waived; we grant 100% waivers to papers whose corresponding authors are based in Research4Life ‘Group A’ countries and 50% waivers to those who are based in ‘Group B’ countries. If you are funded by a grant, your funders should pay.

If your institution is in a slightly poor country, or is a slightly poor university in a not poor country, and you do not have a grant, then open access publishing is more difficult. If you are a member of EHBEA the Article Publishing Charge is reduced (and as membership of EHBEA is not that expensive and of course you love them, then that is certainly worth doing). Otherwise I can grant a small number of fee waivers through our discretionary waiver scheme in cases where there are really no other options. I will try to continue to publish everything that is worth publishing on academic grounds.

The journal has done well. We had over 100 submissions in 2020 (despite the elephant). Thank you to all our authors, reviewers and editors for your work. I am very happy at the diversity of the subject matter. Our authors have included evolutionary-minded anthropologists, archaeologists, biologists, psychologists, linguists, economists and philosophers (and I think at least one lawyer); this makes us a fairly unique home for publishing evolutionary human science. Cultural evolution, in its broadest sense, looms large. We have not been recording the gender or ethnicity of authors, but eyeballing the author names, the gender balance looks good in both senior and junior authors. Our papers are coming from around the world, with some obvious regional gaps. Authors from Africa remain a tiny minority. Awareness that those of us from wealthy countries predominate in the authorship of papers about poorer or less educated countries where we have done research has moved up the agenda. Anthropology is one of several disciplines built on pretty dodgy foundations. I am pleased to note that a wider range of researchers are now being recognised as authors on papers about the research they have contributed to when working, often on fieldwork, with foreign researchers from wealthier countries. Expanding our role as trainers in research beyond our home countries will hopefully diversify our disciplines and help generate more lead authors from under-represented parts of the world. Academic research should not just be for wealthy countries. Of course, diversity within universities in wealthy countries still has a long way to go too – so slow, but I feel academia is at least crawling along in the right direction (even if government policies are not). Several special issues have happened or are in progress, and we may be able to use this route in future to further encourage diversity in our authorship. Do please contact me if you are interested in guest editing a special issue on anything within our remit. Special collections so far have covered TransEurasian Languages and Genes, Children and Innovation, and forthcoming collections will include one on research in South America and a volume to mark the 150th anniversary of the publication of *The Descent of Man* in 2021.

I hope you have had a very happy Christmas holiday and celebrated the New Year in your small gatherings. Let's hope that vaccines will make 2021 look rather different.

